# Thoracolaparoscopic radical esophagectomy for esophageal cancer based on the mesoesophageal theory

**DOI:** 10.1038/s41598-023-35513-w

**Published:** 2023-05-30

**Authors:** Yu-Xiang Sun, Tian-Yu Zhu, Guo-Jun Wang, Bu-Lang Gao, Rui-Xin Li, Jing-Tao Wang

**Affiliations:** Department of Gastrointestinal Surgery, The First Affiliated Hospital of Zhengzhou University, 1 Jianshe Road, Zhengzhou, 450052 China

**Keywords:** Diseases, Gastroenterology, Medical research, Oncology

## Abstract

To explore the feasibility of mesangium or membrane anatomy theory in thoracolaparoscopic radical esophagectomy for esophageal cancer, 98 patients with esophageal cancer were enrolled including 45 patients in the mesoesophageal esophagectomy group and 53 patients in the non-mesoesophageal esophagectomy group. Thoracolaparoscopic radical esophagecotmy was technically successful in all patients. Compared the non-mesoesophageal group, the mesoesophageal group had significantly (P < 0.05) shorter surgical duration (211.9 ± 42.0 min vs. 282.0 ± 44.5 min), less blood loss during the procedure (68.9 ± 45.9 ml vs. 167.0 ± 91.4 ml), more harvested lymph nodes (25.9 ± 6.3 vs. 21.8 ± 7.3), shorter hospital stay after surgery (10.5 ± 2.5 d vs. 12.5 ± 4.2 d), shorter fasting time or quicker postoperative feeding time (7.3 ± 1.2 d vs. 9.5 ± 3.9 d), and quicker removal of the thoracic drainage tube after surgery (7.7 ± 2.0 d vs. 9.2 ± 4.1 d). The overall incidence of postoperative complications was 46.7% (21/45) in the mesoesophageal group, which was significantly (P = 0.02) fewer than that (69.8% or 37/53) of the non-mesoesophageal group (P = 0.020). During follow-up 20.6 ± 4.3 or 20.8 ± 3.4 months after esophagectomy, liver metastasis occurred in 1 case and lung metastasis in 1 in the mesoesophageal group, whereas liver metastasis occurred in 2 cases, mediastinal metastasis in 2, and anastomotic recurrence in 1 in the non-mesoesophageal group. The mesoesophageal group had significantly better physical function (81.9 ± 7.3 vs. 78.3 ± 7.6), social function (65.1 ± 7.1 vs. 56.2 ± 18.2), global health status (65.3 ± 10.1 vs. 58.7 ± 12.4), and pain improvement (29.5 ± 9.5 vs. 35.6 ± 10.6). The overall survival rate was 82.2% (37/45) in the mesoesophageal group and 71.7% (38/53) in the non-mesoesophageal group (P = 0.26). The disease-free survival rate was 77.8% (35/45) for the mesoesophageal group and 62.3% (33/53) for the non-mesoesophageal group (P = 0.13). In conclusion:, the mesangium or membrane anatomy theory can be used safely and effectively to guide thoracolaparoscopic radical esophagectomy for esophageal cancer, with advantages of shorter surgical time, less bleeding, more lymph node harvest, fewer complications, and faster postoperative recovery.

## Introduction

Esophageal cancer is one of the most frequently diagnosed malignancies throughout the world and is the sixth commonest cause of deaths related to cancers^1^. The incidence of this cancer in the world has risen by approximately 50% over the past twenty years^2^. Radical esophagectomy remains the optimal approach of treatment for resectable esophageal malignancies. Nonetheless, traditional open surgical procedures have resulted in massive blood loss as well as high complication rates^3,4^. Because of fast advances in endoscopic technology and wide-spread concept of minimal invasiveness, an increasing number of medical centers have begun to use endoscopic techniques in radical resection of esophageal cancer^1,5–8^. Compared with open surgery, the field of vision is clearer, the operation is minimally invasive and more precise, and better surgical outcomes have been achieved with the endoscopic technique. However, radical esophagectomy has to eliminate possible draining lymphatic systems including lymphatic nodes and tubes near the esophagus and at the mediastina, which may possibly increase surgical trauma, duration, and complication rates even with use of the endoscopic technique for esophagectomy. Radical excision of gastrointestinal cancers with complete removal of organ-specific mesentery has been proposed for total dissection of the original malignant lesion and the relevant lymphovascular drainage system^9–12^. Complete removal of the mesentery and the lymphovascular drainage system within the mesentery can stop cancer cells from dissemination and metastasis through the draining system to other locations. This concept has been applied in total mesorectal excision (TME) and complete mesocolic excision (CME) and has resulted in improved prognoses^9–11^.

However, this mesangium or membrane theory has not been widely accepted in radical esophagectomy for esophageal cancers. The mesoesophagus is a two-layer membranous structure of connective tissue linking the aorta and the left lateral surface of the esophagus, which has been described in previous studies and confirmed in cadaver and imaging research^12–16^. Some studies have applied the knowledge of mesoesophagus in total esophagectomy as in TME and CME, which has been named as mesoesophageal esophagectomy^12,13,17^. In this study, we applied this concept to endoscopic esophagectomy for esophageal cancer with the hypothesis that this approach of mesoesophageal esophagectomy would significantly improve the outcome of esophagectomy.

## Materials and methods

### Subjects

**Figure 1:**
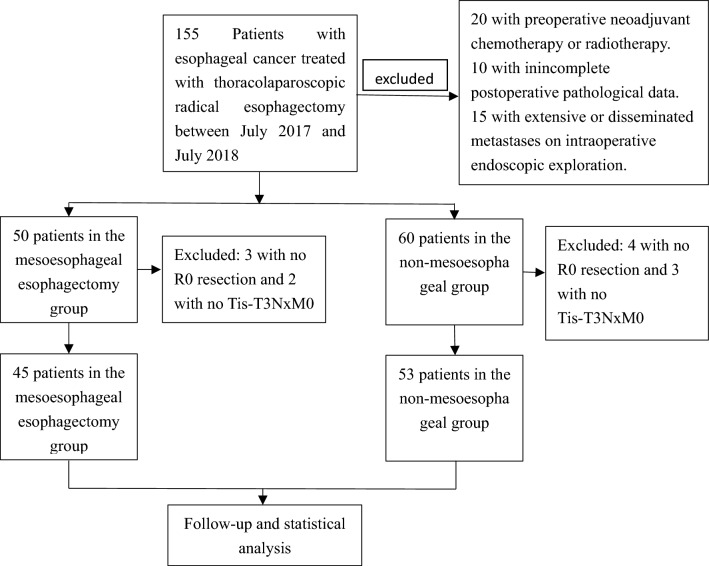
Flow diagram of data collection.

This retrospective one-center case–control study was approved by the ethics committee of the First Affiliated Hospital of Zhengzhou University, and all patients or their family members had provided written informed consent to participate. All methods were performed in accordance with the relevant guidelines and regulations. Patients with esophageal cancer treated with thoracolaparoscopic radical esophagectomy between July 2017 and July 2018 were enrolled (Fig. 1). The inclusion criteria were patients with esophageal cancer confirmed by preoperative endoscopic biopsy, no distal metastasis on thoracic and abdominal medical imaging, treated with thoracolaparoscopic radical esophagectomy and R0 resection confirmed on postoperative pathology, and cancer stage of Tis-T_3_N_x_M_0_ based on the tumor stage according to the TNM score of esophageal cancer in the eighth edition of AJCC^18^. The exclusion criteria were patients with extensive or disseminated metastases on intraoperative endoscopic exploration, preoperative neoadjuvant chemotherapy or radiotherapy or incomplete postoperative pathological data. Patients were enrolled and divided into the mesoesophageal esophagectomy group and non-mesoesophageal esophagectomy group. In the mesoesophageal group, radical mesoesophageal esophagectomy was performed according to the membrane theory, with the mesoesophagus and its contents being completely dissected, including the esophagus, lymph nodes, nerves, blood vessels, and adipose tissues as one intact package. The mesoesophagus indicates the periesophageal connective layers as described in studies of minimally invasive esophagectomy and magnetic resonance imaging^12–16^. In the non-mesoesophageal group, radical esophagectomy was not performed according to the mesoesophageal anatomy.

### Surgical approaches

The surgical procedure for both groups were divided into thoracic, abdominal and neck procedures. In the thoracic operation, thoracic esophageal dissociation and lymph node dissection were performed. In the abdominal operation, the stomach was dissociated, and perigastric lymph node dissection and tubular stomach reconstruction were carried out. In the neck procedure, transection of esophagus and reconstruction of digestive tract were performed.

### Surgery for the mesoesophageal group

#### Thoracic operation (Fig. 2)

**Figure 2:**
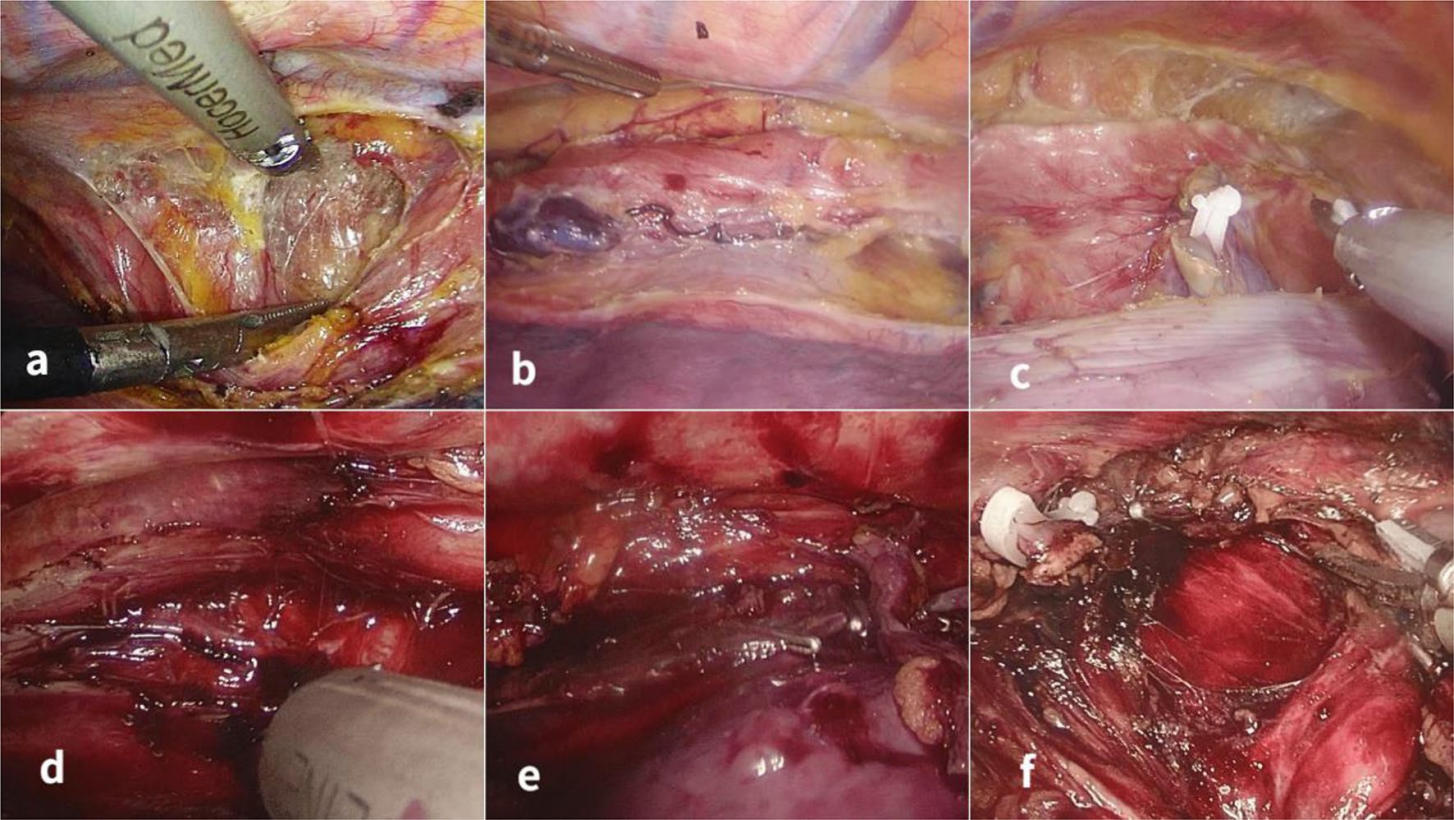
Surgical comparison between two groups. A-C. Throacolaparoscopic radical mesoesophageal esophagectomy. A. During separation between membranes, a fused fascial space was shown to be a white bloodless operation field between the esophagus and esophageal bed. B. The esophagus and lymph nodes were dissociated en bloc according to the membrane separation principle. C. White loose tissue was seen in the fascial space after dissociation of the esophagus, tracheal membrane and esophageal bed. D-F. Throacolaparoscopic non-mesoesophageal radical esophagectomy. D. In dissociation of the posterior mediastinum without adherence to the principle of membrane separation, more blood was present in the operation field. More blood was present when dissociating the esophagus. F. The integrity of esophagus and fascia was destroyed.

The thoracoscopic procedure was performed under general anesthesia with the patient in the prone position using the three trocar technique. After establishment of pneumothorax, the posterior mediastinum was exposed, and the azygos vein was severed. After the esophagus was pressed down, dissection was performed at the obvious intersection of the external esophageal fascia and the thoracic aortic wall to find the fusion space between the esophageal fascia and the thoracic aortic wall. By performing along the aortic wall, the fusion space was expanded to the left until the left mediastinal pleura was reached. The fascia attached to the esophagus was completely separated along the mediastinal pleura, and lymph nodes within the fascia were all dissected. Dissociation was performed downwards to the diaphragmatic hiatus and the cardia and up to the tracheal bifurcation. At the obvious intersection of the extraesophageal fascia and pericardium, the same fusion fascia space was identified to enter the posterior pericardial space. The esophagus was dissociated, and lymph nodes within the esophageal fascia were dissected in the same way. The posterior pericardial space was expanded up to the thoracic inlet and down to the diaphragmatic hiatus to meet the anterior thoracic aortic space. The vagus nerve was disconnected at the right main bronchial membrane, and the esophagus was dissociated along one side of the tracheal membrane to the level of the thoracic inlet. The chest was closed after inserting a thoracic drainage tube.

#### Abdominal procedure (Fig. 3)

**Figure 3:**
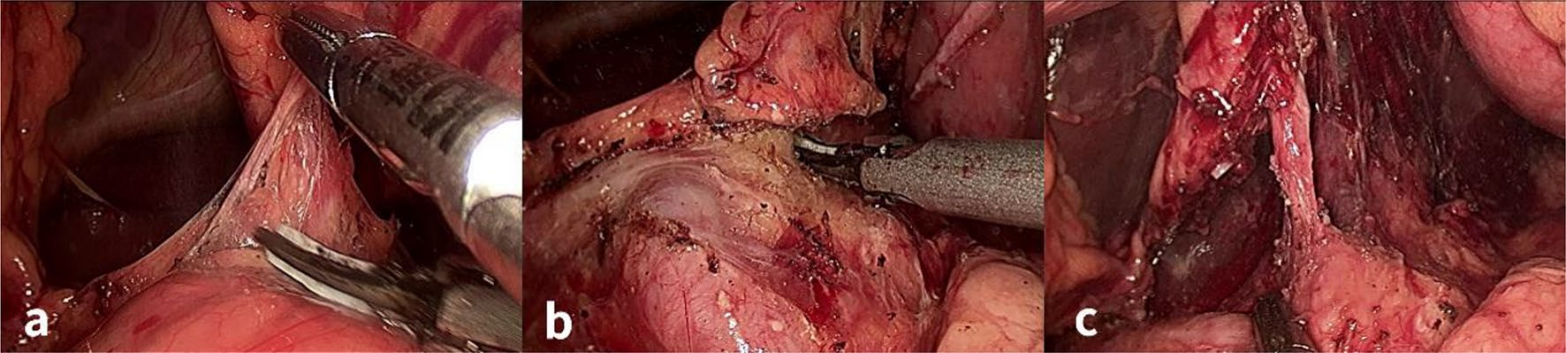
Dissociation of left gastric mesangium in thoracolaparoscopic radical mesoesophageal esophagectomy. A. The left gastric mesangium was lifted and dissociated along the fascial space. B. After separation between membranes, the root of the left gastric vessels was exposed, with the upper left gastric mesangium being kept intact. C. The left gastric vessels were shown after dissociation.

The patient was changed to the supine position, and the five-hole method for laparoscopic surgery was performed. The principle of separation between membranes was followed. The left and posterior gastric mesangium was exposed, and the left gastric blood vessels were disconnected at the root of left gastric mesangium. The lymph nodes were dissected as a whole. The hepatogastric ligament was dissociated to the right diaphragm angle. The diaphragmatic esophageal ligament was disconnected transversely near the diaphragm angle above the cardia, and the left mesentery of the stomach was kept intact before the esophageal hiatus was cut open.

#### Neck procedure

An oblique incision was made on the left of the neck along the front edge of the sternocleidomastoid muscle, and the esophagus was pulled out and disconnected. A 3 cm incision was made in the middle of the upper abdomen, and the esophagus and stomach were pulled out of the abdominal cavity. A tubular stomach was constructed, and the specimen was removed. The tubular stomach was pulled out from the neck incision along the esophageal bed and anastomosed with the proximal end of the esophagus.

### Surgery for non-mesoesophageal group (Fig. 2)

During thoracic operation, the patient took the lateral position, the azygos vein arch was disconnected, and the esophagus was directly dissociated. During the operation, the space of esophageal fascia was not specially searched, the esophagus and surrounding tissues were separated and disconnected, and visible thoracic lymph nodes were cleaned according to the area. After reaching the upper part of the pancreas during abdominal operation, the left mesangium of the stomach was not deliberately dissociated, the stomach was dissociated, and lymph nodes were cleaned in a conventional way. Digestive tract reconstruction was carried out in the neck in the same was as in the mesoesophageal group.

### Quality of life assessment

Postoperative quality of life was assessed according to the European Organization for Research and Treatment of Cancer (EORTC) Quality of Life Questionnaire-Core Questionnaire (EORTC QLQ-C30)^19^ and Esophageal Cancer Module (EORTC QLQ- OES18)^20^. The QLQ-C30 consists of 5 functional scales (physical, role, cognition, emotional, and social function), a global health status and quality of life scale, 3 symptom scales (pain, fatigue, and nausea/vomiting), 6 single items (dyspnea, appetite loss, insomnia, diarrhea, constipation, and financial difficulties). QLQ-OES18 is comprised of 4 scales (dysphagia, reflux, eating, and pain) and 6 single items (trouble swallowing saliva, choking, taste, dry mouth, cough, and speech problems). The scores ranged from 0 to 100. A high score on the functional scale represents a higher level of functionality, whereas a high score on the symptom scale represents severe symptoms.

### Parameters for investigation

Surgical duration, intraoperative bleeding, total postoperative hospital stay, thoracic drainage tube extraction time, postoperative feeding time, number of lymph node dissections, and postoperative complications were recorded. The postoperative complications of the two groups were graded by using the Clavien Dindo complication grading method^21^. Follow-up was performed three months later for the short-term effect of treatment through clinic visit or telephone contact until June 2021.

### Statistical analysis

The statistical analysis was performed with the SPSS software (version 24.0, IBM, Chicago, IL, USA). Measurement data in the normal distribution were presented as a mean ± standard deviation and tested between two groups with the independent t test, and measurement data in the skew distribution were presented as a median and interquartile range and tested with the Rank sum test. Enumeration data were presented as numbers and percentages and tested with the Chi square test or Fisher exact test. Survival analysis was performed with the Kaplan Meier method to plot survival curves, and the Log rank method was applied to test the difference in survival rates between the two groups. The significant statistical P value was set at < 0.05.

## Results

Ninety-eight patients with esophageal cancer were enrolled including 45 patients in the mesoesophageal esophagectomy group and 53 patients in the non-mesoesophageal group (Table 1). No significant (P > 0.05) differences were found in the baseline data between two groups.

**Table 1:** Demography and baseline data of patients (mean ± SD).

Variables	Mesoesophageal group (n = 45)	Non-mesoesophageal group (n = 53)	P
Sex (n, %)			
M	25 (55.6)	32 (60.4)	0.630
F	20 (44.4)	21 (39.2)	
Age (y)	67.1 ± 8.2	66.0 ± 8.1	0.500
Height (cm)	(167.0 ± 7.9)	(166.1 ± 7.9)	0.580
Weight (kg)	(62.2 ± 9.4)	(63.8 ± 9.3)	0.399
BMI (kg/m^2^)	(22.3 ± 2.7)	(23.1 ± 2.9)	0.155
History of smoking	7 (15.6)	13 (24.5)	0.272
Hypertension	14 (31.1)	12 (22.6)	0.344
Diabetes mellitus	6 (13.3)	6 (5.7)	0.190
Pathology(n)			
Squamous cell cancer	44 (97.8)	51 (96.2)	0.53
Adenocarcinoma	1 (2.2)	2 (3.8)	
Cancer location (n, %)			
Upper segment	5 (11.1)	7 (13.2)	0.333
Middle segment	19 (42.2)	29 (54.7)	
Lower segment	21 (46.7)	17 (32.1)	
Tumor T stage (n, %)			
Tis	4 (8.9)	2 (3.8)	0.733
T_1_	9 (20.0)	13 (24.5)	
T_2_	14 (31.1)	16 (30.2)	
T_3_	18 (40.0)	22 (41.5)	
Tumor TNM stage (n, %)			
0	4 (8.9)	2 (3.8)	0.408
I	8 (17.8)	10 (18.9)	
II	13 (28.9)	22 (41.5)	
III	15 (33.3)	17 (32.1)	
IV	5 (11.1)	2 (3.8)	
Nerve invasion	16 (35.6)	11 (20.8)	0.102
Vascular invasion	17 (37.8)	16 (30.2)	0.428

*SD* standard deviation.

Thoracolaparoscopic radical esophagectomy was technically successful in all patients with no patients being converted to open thoracotomy or laparotomy. Compared with patients in the non-mesoesophageal group, patients in the mesoesophageal group had significantly (P < 0.05) shorter surgical duration (211.9 ± 42.0 min vs. 282.0 ± 44.5 min), less blood loss during the procedure (68.9 ± 45.9 ml vs. 167.0 ± 91.4 ml), more harvested lymph nodes (25.9 ± 6.3 vs. 21.8 ± 7.3), shorter hospital stay after surgery (10.5 ± 2.5 d vs. 12.5 ± 4.2 d), shorter fasting time or quicker postoperative feeding time (7.3 ± 1.2 d vs. 9.5 ± 3.9 d), and quicker removal of the thoracic drainage tube after surgery (7.7 ± 2.0 d vs. 9.2 ± 4.1 d) (Table 2). No significant (P > 0.05) difference was found in postoperative albumin reduction (8.0 ± 4.1 g/L vs. 8.9 ± 3.7 g/L).

**Table 2:** Parameters during and after surgery (mean ± SD).

Variables	Mesoesophageal (n = 45)	Non-mesoesophageal (n = 53)	Statistics	P
Surgical time (min)	211.9 ± 42.0	282.0 ± 44.5	t = − 7.974	< 0.001
Surgical bleeding volume (ml)	68.9 ± 45.9	167.0 ± 91.4	t = − 6.527	< 0.001
Postoperative albuminreduction (g/L)	8.0 ± 4.1	8.9 ± 3.7	t = − 1.233	0.221
Lymph nodes harvested (n)	25.9 ± 6.3	21.8 ± 7.3	t = 2.939	0.004
Postoperative hospital stay (d)	10.5 ± 2.5	12.5 ± 4.2	t = − 2.761	0.007
Postoperative feeding time (d)	7.3 ± 1.2	9.5 ± 3.9	t = − 3.620	< 0.001
Days of chest tube extraction (d)	7.7 ± 2.0	9.2 ± 4.1	t = − 2.293	0.024

*SD* standard deviation.

In the mesoesophageal group, anastomotic leakage occurred in 2 cases, massive pleural effusion in 3, and esophagotracheal fistula in 1. In the non-mesoesophageal group, anastomotic leakage occurred in 3 cases, massive pleural effusion in 4, and esophagotracheal fistula in 1. All patients were improved after active drainage or surgical treatment. In the mesoesophageal group, recurrent laryngeal nerve paralysis occurred in 1 case, chylothorax in 1, gastric acid reflux in 4, and atelectasis in 8. In the non-mesoesophageal group, recurrent laryngeal nerve paralysis took place in 3 cases, chylothorax in 2, gastric acid reflux in 4, and atelectasis in 13. All complications were relieved after conservative treatment.

Postoperative pulmonary infection occurred in 1 case in the mesoesophageal group but in 6 cases in the non-mesoesophageal group, but was improved after treatment with antibiotics. Massive intraoperative bleeding occurred in 1 case in the non-mesoesophageal group and was treated with hemostasis and blood transfusion. Clavien-Dindo grade I-II complications occurred in 17 cases in the mesoesophageal group and 30 cases in the non-mesoesophageal group, and Clavien-Dindo grade III or above complications occurred in 4 cases in the mesoesophageal group and 7 cases in the non-mesoesophageal group (Table 3).

**Table 3:** Complications in two groups (n, %).

Variables	Mesoesophageal (n = 45)	Non-mesoesophageal (n = 53)	Statistics	P
Complications (n, %)	21 (20.0)	37 (41.5)	χ^2^ = 5.397	0.020
Anastomotic fistula	2 (4.4)	3 (5.7)		
Massive pleural effusion	3 (6.6)	4 (7.5)		
Recurrent laryngeal nerve paralysis	1 (2.2)	3 (5.7)		
Chylothorax	1 (2.2)	2 (3.8)		
Esophagotracheal fistula	1 (2.2)	1 (1.9)		
Massive bleeding during surgery	0	1 (1.9)		
Pulmonary infection	1 (2.2)	6 (11.3)		
Atelectasis	8 (17.8)	13 (24.5)		
Gastric acid reflux	4 (8.8)	4 (7.5)		
Clavien-Dindo grade				
I	7	11	χ^2^ = 0.331	1.000
II	10	18		
IIIa	3	5		
V	1	2		

The overall incidence of postoperative complications was 46.7% (21 / 45) in the mesoesophageal group, which was significantly (P = 0.02) fewer than that (69.8% or 37 / 53) in the non-mesoesophageal group (P = 0.020).

Follow-up was performed for 20.6 ± 4.3 months in the mesoesophageal group and 20.8 ± 3.4 months in the non-mesoesophageal group. At follow-up, liver metastasis occurred in 1 case and lung metastasis in 1 in the mesoesophageal group. In the non-mesoesophageal group, liver metastasis occurred in 2 cases, mediastinal metastasis in 2, and anastomotic recurrence in 1. During the follow-up, there was no death within 30 days after surgery in both groups.

At 12-month follow-up, one patient died, one patient experienced recurrent esophageal cancer, and 43 patients were finally evaluated with the postoperative quality of life scores using the QLQ-C30 and –OES18 scales in the mesoesophageal esophagectomy group. In the non-mesoesophageal group, two patients experienced recurrence of the esophageal cancer within one year, and 51 patients were entered for the quality of life assessment (Tables 4 and 5).

**Table 4:** Postoperative quality of life scores using QLQ-C30 (mean ± SD).

Variables	Mesoesophageal (n = 43)	Non-mesoesophageal (n = 51)	Statistics	P
Physical function	81.9 ± 7.3	78.3 ± 7.6	t = 2.293	0.024
Role function	70.2 ± 13.9	70.9 ± 8.7	t = − 0.323	0.748
Emotional function	69.0 ± 13.2	72.1 ± 11.6	t = − 1.197	0.235
Cognitive function	82.9 ± 13.4	77.8 ± 12.3	t = 1.950	0.054
Social function	65.1 ± 7.1	56.2 ± 18.2	t = 3.208	0.002
Global health status	65.3 ± 10.1	58.7 ± 12.4	t = 2.869	0.005
Fatigue	33.3 ± 4.8	34.3 ± 13.3	t = − 0.490	0.626
Nausea/vomiting	24.4 ± 8.4	25.8 ± 9.6	t = − 0.744	0.459
Pain	29.5 ± 9.5	35.6 ± 10.6	t = − 2.951	0.004
Dyspnea	34.9 ± 19.1	35.9 ± 9.1	t = − 0.333	0.740
Insomnia	31.8 ± 14.5	32.7 ± 8.1	t = − 0.346	0.731
Appetite loss	32.6 ± 19.9	35.3 ± 7.9	t = − 0.846	0.401
Constipation	25.6 ± 14.2	30.7 ± 11.2	t = − 1.915	0.059
Diarrhea	22.5 ± 15.8	21.6 ± 16.1	t = 0.276	0.783
Financial difficulties	41.1 ± 14.3	40.5 ± 13.8	t = 0.194	0.847

*QLQ-C30* quality of life questionnaire version 3.0, *SD* standard deviation.

**Table 5:** Postoperative quality of life scores using QLQ-OSE18 (mean ± SD).

Variables	Mesoesophageal (n = 43)	Non-mesoesophag eal (n = 51)	statistics	P
Dysphagia	9.6 ± 11.8	10.9 ± 13.4	t = − 0.507	0.613
Eating	16.8 ± 12.9	19.6 ± 13.1	t = − 1.045	0.299
Reflux	22.4 ± 11.2	24.2 ± 11.0	t = − 0.738	0.462
Pain	18.3 ± 11.3	25.1 ± 10.8	t = − 2.925	0.004
Trouble swallowing saliva	4.9 ± 8.2	5.7 ± 9.5	t = − 0.409	0.684
Choking	12.1 ± 12.1	13.7 ± 17.0	t = − 0.510	0.611
Dry mouth	19.9 ± 12.5	20.5 ± 12.6	t = − 0.224	0.823
Taste	10.6 ± 12.1	10.9 ± 12.9	t = − 0.115	0.908
Cough	15.0 ± 13.1	18.5 ± 11.9	t = − 1.372	0.173
Speech	4.5 ± 7.0	4.8 ± 6.8	t = − 0.207	0.836

*QLQ-OSE18* quality of life questionnaire esophageal cancer module,* SD* standard deviation.

Compared with the QLQ-C30 data in the non-mesoesophageal group (Table 4), the mesoesophageal group had significantly better physical function (81.9 ± 7.3 vs. 78.3 ± 7.6), social function (65.1 ± 7.1 vs. 56.2 ± 18.2), global health status (65.3 ± 10.1 vs. 58.7 ± 12.4), and pain improvement (29.5 ± 9.5 vs. 35.6 ± 10.6). No significant (P > 0.05) differences were found in the other parameters. Compared with the QLQ-OES18 data in the non-mesoesophageal group (Table 5), the pain in the mesoesophageal group was significantly (P < 0.05) improved (18.3 ± 11.3vs. 25.1 ± 10.8). No significant (P > 0.05) differences were detected in the other parameters.

**Figure 4:**
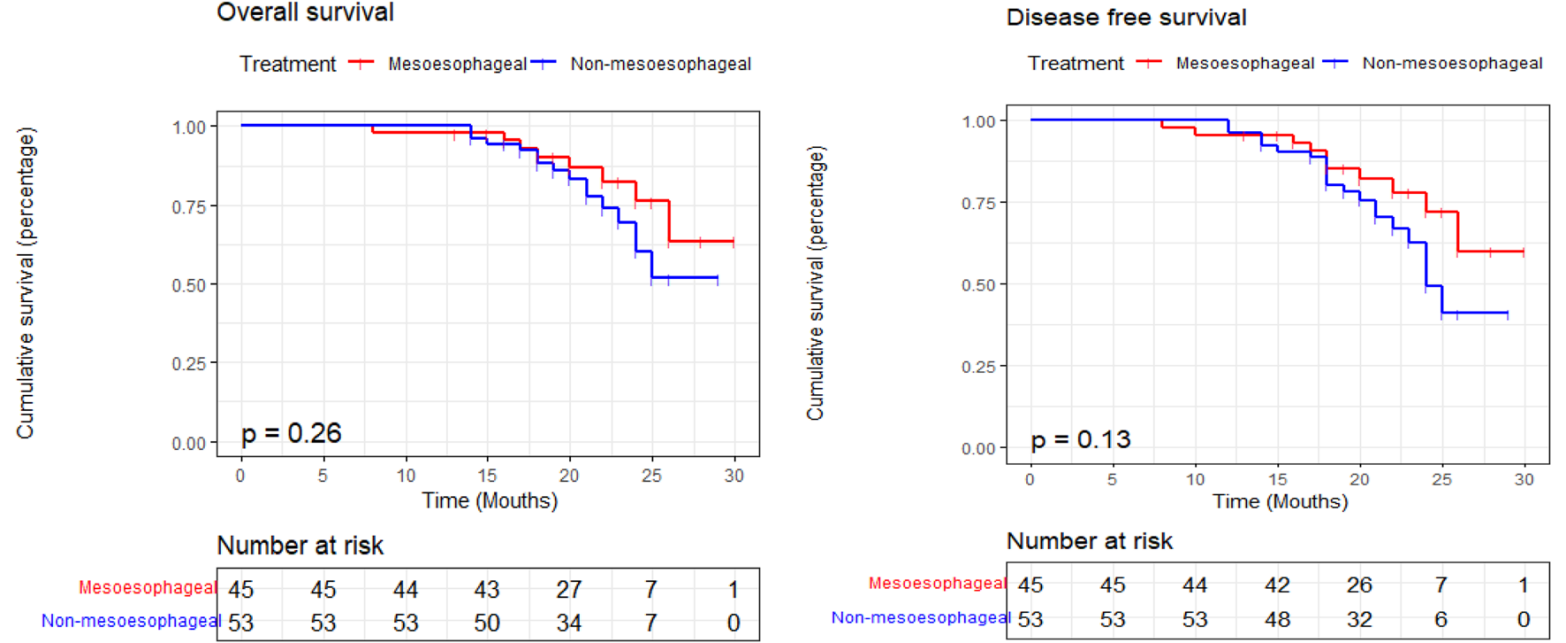
The Overall survival and disease-free survival of patients of esophageal cancer treated with the mesoesophageal or non-mesoesophageal radical esophagectomy.

In the final follow-up of 20.6 ± 4.3 months in the mesoesophageal group and 20.8 ± 3.4 months in the non-mesoesophageal group, the overall survival rate was 82.2% (37/45) in the mesoesophageal group and 71.7% (38/53) in the non-mesoesophageal group, with no significant difference (P = 0.26, Fig. 4). The disease-free survival rate was 77.8% (35/45) for the mesoesophageal group and 62.3% (33/53) for the non-mesoesophageal group (P = 0.13, Fig. 4).

## Discussion

This study investigated the feasibility, effects and complications of membrane theory in thoracolaparoscopic radical esophagectomy for esophageal cancer. It was found that the mesoesophageal theory could be used safely and effectively to guide thoracolaparoscopic radical esophagectomy for esophageal cancer, with the advantages of shorter surgical time, less bleeding, more lymph nodes harvested, fewer complications, and faster postoperative recovery.

Compared with traditional open esophagectomy, endoscopic radical esophagectomy has the advantages of less intraoperative bleeding, lower incision infection and lower local recurrence rates^1^. In order to achieve the ideal surgical effect, the key to successful cancer dissection is to achieve real R0 resection and avoid cancer leakage or residue. Therefore, it is necessary to further optimize the surgical concept and surgical details. The TME theory was proposed in 1986 by Heald et al.^10^ in total resection of rectal cancer and had resulted in better outcomes because the TME theory focuses on complete removal of the malignant lesion together with the primary lymphatic and vascular draining system in the mesorectum as one intact package, including the cancerous lesion, regional lymph nodes, blood vessels, and adipose tissues within the mesorectum. The CME was proposed by Hohenberger et al. in 2009 to resect the primary cancer and relevant draining lymph nodes and vessels within the mesocolon as one intact package^11^. Both the TME and CME surgical techniques have greatly reduced local cancer recurrence and improved the prognosis.

Gong et al.^22–24^ had proposed a mesangium or membrane anatomy theory and the fifth metastasis theory of tumor and considered the mesangial and fascial structures around the digestive tract as an "envelope" structure. According to the theory of mesangium, all internal organs have a generalized mesangium and mesangial bed. During embryonic development, part of the mesangium and the serosa of the mesangial bed fuse and degenerate to form a fused fascia space. Radical tumor resection must adhere to the principle of separation between membranes, dissecting and dissociating along the fascia space. The integrity of the medial mesangium of the space should be maintained to avoid cancer leakage while at the same time maintaining the integrity of the mesangial bed from additional injury^23^. In a word, the tumor lesion together with the surrounding connective tissue, blood vessels, nerves, and lymph nodes within the mesangium should be dissected as a whole so as to reduce the probability of intramesenteric metastasis of cancer. They consequently applied this theory in radical gastrectomy, resulting in good curative effects^22,23^. In clinical practice, some researchers have unconsciously applied mesoesophageal or en bloc esophagectomy for radical treatment of esophageal cancer^12,13,17^, which is in line with the mesangium theory.

From the perspective of embryology, esophagus and stomach are organs developed from the foregut^15^, which may also be used to guide radical esophagectomy. According to the theory of mesangium, the generalized mesangium has the characteristics of common structure, different morphology and widespread existence. Blood vessels and lymph nodes exist only in the mesangium. According to this principle, in radical esophagectomy, it is also necessary to follow the mesangium theory, adhere to separation between membranes, and maintain the integrity of the primary fascia inside the fusion fascia space so as to avoid cancer leakage and reduce bleeding. At the same time, the integrity of the lateral mesangial bed of the gap should be maintained to avoid additional injury^23^.

In our study, the radical esophagectomy in the mesoesophageal group was performed according to the principle of separation between two membranes so as to maintain the integrity of fascia or serosa on both sides of fascial space, whereas that in the non-mesoesophageal group did not adhere to the principle of membrane separation in the fascia space, with dissection and dissociation through traditional surgical techniques. The fascial space was not deliberately pursued for dissociation between two membranes. In comparison of the outcomes between two groups, the mesoesophageal group was significantly better than the non-mesoesophageal group in terms of intraoperative bleeding, surgical time, numbers of lymph nodes harvested, and total incidences of complications (P < 0.05). In adherence to the principle of separation between membranes during mesoesophageal esophagectomy, a clear fusion fascial space can be clearly demonstrated under the highly-magnified endoscope between the inherent fascia of the esophagus and the esophageal bed, and the white bloodless surgical field can also be displayed under the endoscope. Performance in the abdominal cavity is also based on the principle of separation between membranes. Our previous study has revealed that the left gastric lymph node is a high-risk area for esophageal cancer metastasis^25^. Clamping the severed blood vessel at the root of the left gastric mesangium is in line with the principle of "en bloc resection". However, traditional radical esophagectomy does not adhere to the principle of membrane separation, which is bound to destroy the integrity of the fascia on both sides of the fusion fascial space, resulting in more bleeding, unclear visual field, damage to the visceral proper fascia, increases of cancer cell leakage, and damage to the parietal fascia (causing additional injury)^23,24^. No significant differences were found in cancer recurrence or metastasis during follow-up. Even though more cases and longer follow-up are necessary to confirm the outcome, mesoesophageal radical esophagectomy abides by the gastroenterological mesangial theory which adopts the en bloc radical resection. In the en bloc radical resection, the malignant lesion together with the primary lymphatic and vascular draining system in the mesangium, including the cancerous lesion, regional lymph nodes, blood vessels and adipose tissues, is completely removed, resulting in reduction of local cancerous recurrence.

Our study showed that the mesangium theory could be used to guide radical esophagectomy, and adherence to the principle of membrane separation for anatomical dissociation was in line with the principle of "en bloc resection", leading to increased numbers of lymph nodes harvested, reduced bleeding and injury, faster recovery, and better surgical effects.

Some limitations existed in this study, including the retrospective and one-center study nature, a small cohort of patients, Chinese patients enrolled only, no randomization, shorter follow-up, which may all affect the outcome and the generalization. Future prospective, randomized, controlled studies involving a large cohort of patients, multiple centers and longer follow-up are necessary to resolve all these issues for better outcomes.

In conclusion, the mesoesophageal theory can be used safely and effectively to guide thoracolaparoscopic radical esophagectomy for esophageal cancer, with the advantages of shorter surgical time, less bleeding, more lymph node harvest, fewer complications, and faster postoperative recovery. Further studies with a longer follow-up are necessary to confirm these effects.

## Data Availability

The datasets generated and/or analysed during the current study are not publicly available because these datasets are owned by our hospital and are not allowed to be made publically available but are available from the corresponding author on reasonable request.
